# The Many Faces of *Enterococcus* spp.—Commensal, Probiotic and Opportunistic Pathogen

**DOI:** 10.3390/microorganisms9091900

**Published:** 2021-09-07

**Authors:** Beata Krawczyk, Paweł Wityk, Mirosława Gałęcka, Michał Michalik

**Affiliations:** 1 Department of Molecular Biotechnology and Microbiology, Faculty of Chemistry, Gdańsk University of Technology, ul. Narutowicza 11/12, 80-233 Gdańsk, Poland; pawel.wityk@pg.edu.pl; 2 Institute of Microecology, ul. Sielska 10, 60-129 Poznań, Poland; drgalecka@instytut-mikroekologii.pl; 3 Medical Center MML, ul. Bagno 2/E/4, 00-112 Warsaw, Poland; michalim@mml.com.pl

**Keywords:** *Enterococcus* spp. probiotics, application, risk factors, virulence, antibiotic resistance

## Abstract

*Enterococcus* spp. are Gram-positive, facultative, anaerobic cocci, which are found in the intestinal flora and, less frequently, in the vagina or mouth. *Enterococcus faecalis* and *Enterococcus faecium* are the most common species found in humans. As commensals, enterococci colonize the digestive system and participate in the modulation of the immune system in humans and animals. For many years reference enterococcal strains have been used as probiotic food additives or have been recommended as supplements for the treatment of intestinal dysbiosis and other conditions. The use of *Enterococcus* strains as probiotics has recently become controversial due to the ease of acquiring different virulence factors and resistance to various classes of antibiotics. Enterococci are also seen as opportunistic pathogens. This problem is especially relevant in hospital environments, where enterococcal outbreaks often occur. Their ability to translocate from the gastro-intestinal tract to various tissues and organs as well as their virulence and antibiotic resistance are risk factors that hinder eradication. Due to numerous reports on the plasticity of the enterococcal genome and the acquisition of pathogenic microbial features, we ask ourselves, how far is this commensal genus from acquiring pathogenicity? This paper discusses both the beneficial properties of these microorganisms and the risk factors related to their evolution towards pathogenicity.

## 1. Introduction

Enterococci are a diverse, species-rich group of lactic acid bacteria isolated from various environments, including from the digestive systems of humans, animals, and insects but also from natural biomes such as water [1,2], sewage [3], soil [4], and arable land [5]. Enterococci have also been isolated from plants such as olives [6] and are found on plants in the wild [7,8]. Some enterococci species are commensal, can stimulate the immune system, and have a significant influence on the maintenance of intestinal homeostasis [9,10]. Enterococci can be used as a factor to support the immune system in the form of a probiotic (diet supplement or therapeutic application). Likewise, enterococci play a role in food technology as the initiating culture involved in the fermentation of meats and cheeses [11] and the preservation of food [12,13,14,15]. On the other hand, enterococci can act as pathogens [16]. They are responsible for food contamination [12] and due to their sometimes present virulence and multi-drug resistance, they pose an epidemic threat in the hospital environment [17]. Research suggests they may have a role in the development of colon tumorigenesis as well [18]. Some countries disregard enterococci regardless of their positive features, while others accept them despite being a threat in certain situations. The approach to enterococci differs from country to country, so it is important to standardize the criteria that allow a strain to be considered beneficial for health. The advantages and disadvantages of enterococci applications are described in this review.

## 2. Enterococci as Commensal Microorganisms and Their Influence on the Immune System

Human microbiome research has shown that the body is inhabited by approximately 5000 species of microorganisms belonging to 2000 genera and 25 phyla, which possess a total of 316 million genes [19]. It is estimated that there are 9 million different genes of bacterial origin related to the human digestive system alone [20]. Based on the Gene Catalog (IGC) of the human gut microbiome and mapping to the eggNOG database [21], approximately 40% of all genes are unknown or have an undefined function.

Genomic analysis based on the 16S rRNA sequencing of 202 complete human gut bacteria genomes estimates the qualitative and quantitative composition of human microbiota [20]. The gastrointestinal (GI) tract is mainly colonized by species belonging to the phylum Firmicutes, which accounts for up to 65% of all bacteria. The next biggest phylum is Bacteroidetes, which comprises 30%, with the remaining 5% being Proteobacteria and Actinobacteria [20].

The development of metagenomic tools has revolutionized our understanding of the gastrointestinal microbiome and its symbiotic relationship. Metagenomic approaches on the study of the human microbiome has enhanced the ability to understand how the gut microbiota is influenced by various long-term diets, geographical locations, age, and disease [22,23,24]. The European project MetaHIT23 and the American Human Microbiome Project are based on fecal metagenomic analyses and distinguish three main robust clusters named “enterotypes,” including the genera Bacteroides (enterotype 1), Prevotella (enterotype 2), and Ruminococcus (enterotype 3). Specific species composition enterotypes in the gut microbiome are stable, but their abundance and proportions vary between individuals [22,25]. Enterotypes differ in composition at the phylum, genus, and gene level, along with their abundance of cohabiting genera. Moreover, metagenomic analyses of fecal samples confirms that the Firmicutes and Bacteroidetes phyla constitute the vast majority of the dominant human gut microbiota [25,26,27].

Enterococci belong to the phylum Firmicutes in the family Enterococcaceae, which includes a great variety of species. Enterococci are a natural component of the human microbiota. They colonize the lower GI tract, the oral cavity, and the genital tract [28]. There are approximately 10^6^ to 10^7^ *Enterococcus* in the human intestine (<1% found in the ileum, up to 1% in the colon [22]), most of which are either *E. faecalis* (10^5^–10^7^ CFU/gr feces) or *E. faecium* (10^4^–10^5^ CFU/gr feces). In addition to *E. faecalis* and *E. faecium* and *E. cecorum* and *E. durans* are also frequently isolated [29], while *E. caseliflavus*, *E. hirae*, *E. gallinaroum*, and *E. avium* are occasionally detected [30].

As commensal bacteria, they participate in the metabolism of nutrients (carbohydrates, lipids, and proteins) to maintain the pH of the environment in which they live, synthesize vitamins and other metabolites that are important for normal functioning, prevent the binding and spread of putrefactive bacteria, and have an impact on the human immune system, i.e., humoral and cellular immunity [31]. Since enterococci are present in significant numbers in the human microbiome, this suggests they may play a significant role in the digestive tract. The colonization of the digestive system is a dynamic process; however, it depends on many factors, including genetic aspects, maternal microbiota, type of delivery, environmental conditions, and diet [32].

Enterococci (*E. faecalis*, and *E. faecium* to a lesser extent), along with *Bifidobacterium*, *E. coli* and *Lactobacillus*, colonize the digestive system of most healthy breastfed infants in the first 7–10 days after birth [33,34]. These microorganisms mainly come from the physiological flora of the mother’s genital tract; however, enterococci also dynamically colonize the gastrointestinal tract of newborns born by cesarean section. Natural factors facilitate their ability to colonize and survive at a pH of 9.6. Moreover, these species display resistance to bile salts, which allow them to overcome the human digestive system and colonize the large intestine [35,36]. Various enterococcal species are also acquired in adulthood from certain foods, such as pork, poultry, and ripening rennet cheeses [30,37].

It is widely considered that without bacteria, there is no functioning immune system, as they are responsible for stimulating the immune system of the intestinal mucosa [10]. The gut microbiota is seen as a virtual endocrine organ [38], and microbes that are permanently associated with the gut microbiota are regarded as commensals [39]. *E. faecalis* plays an immunomodulatory role and is responsible for the activation of CD4, CD8 (CD-cluster of differentiation) cells, and B lymphocytes.

The GI tract has developed many defense mechanisms to control the gut microbiota. The intestinal lymphatic system (gut-associated lymphoid tissue) is the immune organ responsible for the production of secretory immunoglobulin A (sIgA). sIgA is an important element of the intestinal barrier, as it prevents the adhesion of microorganisms to the epithelium, neutralizes bacterial toxins, coats and agglutinates microorganisms, and has a bacteriostatic effect. The intestinal barrier is also shaped by enterocytes that form strong adherens junctions (AJs) (zonulae occludentes and tight junction). When these junctions are loosened, the problem of a so called “leaky gut” arises. Such relaxed junctions allow bacteria to pass through and initiate an immunological cascade. Enterococci, especially *E. faecium* and *E. faecalis*, subsequently cross the intestinal barrier, which can lead to bacteremia/sepsis in patients [40]. Therefore, maintaining the microbial balance in the gut is of utter importance. This maintenance especially applies to patients with blood cancer, as immunosuppressive drugs or antibiotic therapy change the intestinal environment and its permeability, facilitating the translocation of bacteria into the blood bed [41,42].

## 3. Enterococci as Probiotics

According to the Food and Agriculture Organization of the United Nations and World Health Organization, probiotics are live microorganisms identified at the strain level which, when given in an appropriate amount, have a beneficial effect on the health of the host [43]. They are often interpreted as “live biotherapeutics” for human use [44] and “direct-fed microbials” in animal feeds [45,46].

Probiotics can be single or multi-species, as some theorize that a mixture of probiotic bacteria not only interact or compete, but also influence each other’s beneficial effects. This interaction means that while using each bacterium separately can yield results, taking them together may be less effective or not effective at all, highlighting the clinical significance of the relationship between bacterial species. Probiotics must meet certain requirements; for example, they should be isolated from the hosts that they are intended to be used for, should be able to survive in the GI tract, and should produce compounds with bacteriostatic activity. According to the Food and Drug Administration, probiotic bacteria should “Generally be Recognized as Safe” [47]. In Europe, a “Qualified Presumption of Safety” is responsible for recommending biological agents intentionally added to food or feed, and information is available in the European Food Safety Authority scientific panels [48].

**Figure 1:**
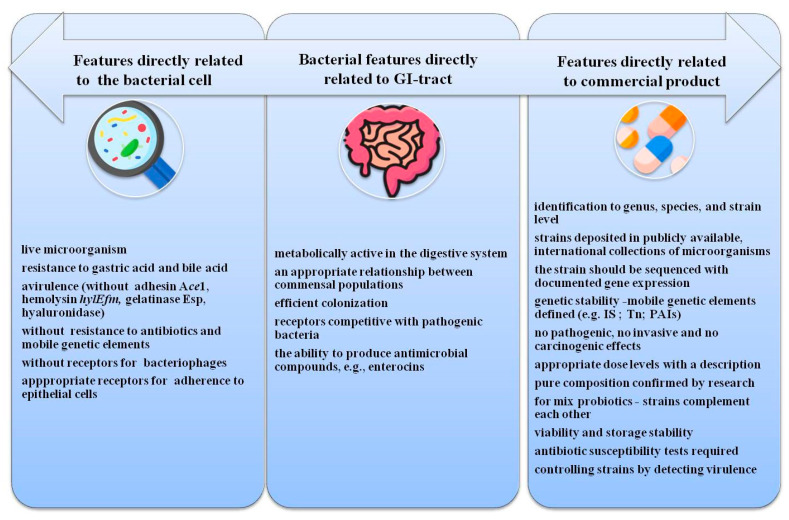
Required features of *Enterococcus* spp. in relation to probiotics.

The features describing a probiotic are shown in Figure 1.

### 3.1. Enterococcal Probiotic Strains

The use of enterococci in the treatment of various diseases, such as chronic and recurrent infections of the upper respiratory tract, skin lesions, or chronic diseases related to the sinuses (chronic sinusitis), was first described in the 1950s [49]. The first probiotic therapies, which entailed the application of *Enterococcus faecalis* following antibiotic therapy, was described by Heinz Kolb in 1955 [49]. Currently, probiotic preparations of *E. faecalis*, sometimes enriched with *Escherichia coli* and lactobacilli, are recommended for the treatment of diseases such as urinary tract inflammation, sinusitis or bronchitis, and the common cold. In Germany, *Enterococcus faecalis* (DSM 16431) is sold as a drug under the brand name Symbioflor 1 (SymbioPharm, Herborn, Germany) and is recommended for acute and recurrent sinusitis or bronchitis [13,50]. Likewise, this non-pathogenic probiotic bacterium has been fully sequenced, and the genome sequence has been deposited in the European Molecular Biology Laboratory database under the accession number HF558530. The circular genome (2,810,675 bp), with 37.72% GC content, consists of 2733 coding sequences and 63 tRNAs.

Clone DSM 16431 carries 2 large mutations which eliminate the *van*B operon and genes encoding virulence factors such as cytolysin L, gelatinase, hyaluronidase, bacteriocin, and *efaA* surface proteins. The genome contains a unique bacteriophage region (1,846,700–1,891,973) [51,52] as well. This strain contains features to facilitate its colonization in the digestive system. Specifically, the *agg* gene encodes an aggregating factor and facilitates gut colonization, while the *esp* and *ace* genes enhance adhesion and colonization. Additional features involved the ability to survive against acids (gastric acid) and proliferation within the intestinal epithelium [53]. Finally, a crucial property of this strain is its lack of any antibiotic resistance mechanisms.

The genome of *E. faecalis* Symbioflor 1 was compared to the first vancomycin resistant strain *E. faecalis* V583 isolated in the United States and has been completely sequenced (Accession No. NC_004668) [54]. *E. faecalis* Symbioflor 1 does not contain any pathogenic features or antibiotic resistance genes previously identified in *E. faecalis* V583, such as cytolysin, enterococcal surface protein, gelatinase, hyaluronidase, or the peptide antibiotic AS-48. These enterococcal virulence factors have been recognized as suitable markers for the risk assessment of strains used in food products or probiotics [54].

Baccouri et al. recently [55] described two new strains of *E. faecalis*, OB14 and OB15, which were isolated from traditional Tunisian fermented dairy products, Testouri and Rigouta cheese, respectively. Genomic sequencing revealed that OB15 is genetically related to the *E. faecalis* Symbioflor 1 (DSM 16431) and displays potential as a probiotic, while the second OB14 strain is characterized by tetracycline resistance and high virulence due to the presence of the cytolysin gene. In another study [56], transcriptomic analysis of several clinical strains isolated from the urinary tract of patients was performed and compared to the probiotic strain *E. faecalis* Symbioflor 1. In particular, energy and nitrogen metabolism, cell stress, and metal acquisition were compared. Citrate and aspartate were important for the growth of both *E. faecalis* groups in urine, and related gene expression was similar in both groups. According to the authors, virulence factors are responsible for adaptation to an ecological niche and ultimately determine the pathogenic potential of bacteria.

*E. faecalis* is not alone in having probiotic properties; *E. lactis* [57], *E. hirae* [58], *E. durans* [59], and *E. faecium* [60] are also used as probiotics. The purpose of using these preparations is to improve the composition of the intestinal microbiota [61,62]. The probiotic strain *E. faecium* M-74 (Aberdeen, UK; the National Collections of Industrial Food and Marine Bacteria (NCIMB) registered no 11181) was isolated from the gastrointestinal tract of healthy Swedish children and exhibits immunomodulatory, antimutagenic [63,64,65], and hypocholesterolemic properties [66]. *E. faecium* strain 11181 is also currently used in animal feed as a supplement [67].

Other probiotic preparations exist and are recommended for the treatment of irritable bowel symptoms. The “Bioflorin” preparation includes one of the first cultured probiotic *E. faecium* SF68^®^ strains [60]. *E. faecium* SF68^®^ (the strain deposit of *Enterococcus faecium* NCIMB 10415 in Aberdeen, Scotland, registered trademark owned by Cerbios-Pharma SA) displays a wide clinical application for the treatment digestive tract disorders in humans [68]. Currently *E. faecium* SF68^®^ is recommended for veterinary applications as a probiotic supplement (e.g., FortiFlora^TM^). *E. faecium* SF68^®^ has been described to prevent and treat diarrhea in pets and cats [69,70]. Probiotics such as Cylactins (Hoffmann-La Roche, Basel, Switzerland) and 85 Fargo 688s (Quest International, Naarden, The Netherlands) with *E. faecium* are also used for veterinary applications.

### 3.2. The Probiotic Importance of Enterococcus spp. and Applications

Pregnant women, newborns, and the elderly are typically at greater risk of infection due to undeveloped or weakened immune systems. Research has demonstrated that supplementation with probiotics in the elderly leads to the growth of potentially beneficial intestinal bacteria but also leads to the increased activation of a non-specific immune response [71]. In some Western European countries, a fermented milk drink called “Gaio” (yogurt) is available, containing bacteria called “Causido,” which includes the *E. faecium* K-77D strain and two strains of *Streptococcus termophilus*. These bacteria come from the intestinal microbiota of elderly people living in Abkhazia in the Caucasus, an area known for longevity amongst its population [72].

Microbiota are important in maintaining the physiological balance of the intestine and have a significant role in immune homeostasis. Enterococci produce small peptides that belong to the bacteriocin group and have antimicrobial properties. These include enterocin A, B, P, ON-157 produced by *E. faecium*, and L50 made by *E. faecalis* [53]. Enterocins exhibit broad antimicrobial activity, inhibiting the multiplication of bacteria such as *Staphylococcus* spp., *Bacillus cereus*, *Listeria monocytogenes*, *Clostridium* spp., *E. coli*, *Pseudomonas aeruginosa*, and *Vibrio cholera* [73]. *E. faecalis* KT11, isolated from Kargı Tulum cheese, produces a bacteriocin with antimicrobial activity against Gram-positive (*L. monocytogenes*, *S. aureus*, *B. subtilis*) and Gram-negative (*P. aeruginosa*, *K. pneumoniae*, *S. marcescens* and *E. aerogenes*) bacteria and inhibits the growth of methicillin- and/or vancomycin-resistant bacteria [74].

Enterococci (e.g., *E. faecium* M-74, *E. durans* KLDS) are also characterized by their ability to lower cholesterol levels [63,75]. These bacteria produce a hydrolase which catalyzes the bile acid deconjugation process and assists in cholesterol integration into the bacterial cell wall or assists in precipitation if the environment is acidic [63,75]. Mego et al. demonstrated the use of the probiotic *E. faecium* M-74 in the treatment of gastrointestinal complications in patients with myeloid leukemia [66]. Another scientific report by Viaud et al. in a mouse model showed that *Enterococcus hirae* helps shape the anti-cancer immune response. [76]. The authors showed that cyclophosphamide (one of the drugs that stimulates anti-cancer immune response) changes the composition of the microbiota in the small intestine and induces the translocation of certain Gram-positive bacteria, including *E. hirae*, to the secondary lymphoid organs [76].

The importance of probiotic strains has been confirmed not only in humans, but also in animals. Benyacoub et al. [77] confirmed an immunomodulatory role of *E. faecium* SF68 on the intestinal mucosa and the development of the digestive system in young dogs. Another preparation, Cylactin^®^, containing the strain *E. faecium* NCIMB 10415, has been used in pig and poultry farming as a feed additive in European Union countries instead of supplementary avoparcin to stimulate animal growth [46,48,78].

Enterococci can be found in food products such as untreated milk, cheese, meat [15,46], and plant products (fermented vegetables) [79]. Their presence in some products is considered desirable. Enterococci are mainly used for the production of regional foods in Mediterranean countries. In the food industry, selected enterococcal strains contribute to the improvement of the aroma, texture, and taste of fermented dairy products [13,80]. Probiotic strains can degrade proteins into peptides and amino acids, break down citrates, and produce aromatic substances with lipolytic and proteolytic properties. They are used as starter cultures in dairy products due to their ability to conduct the proteolysis, lipolysis, and metabolism of citrate and pyruvate [14,15]. Furthermore, enterococci produce substances such as acetaldehyde, acetoin, diacetyl, or 2,3-butanediol. In addition, these bacteria inhibit the proliferation of spoilage microbes by producing enterocins. Hence, bacteriocins can be used as food preservatives [12]. Due to their resistance to thermal treatment (cooking, pasteurization, or fermentation), they can be used as a hygienal indicator in food production as well. According to European guidelines, the producer is responsible for the safety of probiotics and starter strains. Producers therefore have an obligation to evaluate them to be safe for use.

## 4. *Enterococcus* spp. as Opportunistic Pathogens

### 4.1. Hospital-Acquired Infection

Enterococcal colonization is observed 10–20 times more often than the symptoms of infection [81], but some epidemiological studies conducted on carriers indicate a possible association between colonization and symptomatic infection [82]. Enterococci are opportunistic pathogens that, outside of their typical commensal habitats (GI tract), may be the cause of various infections (urinary tract infections, sepsis, bacteremia, and endocarditis) [83,84,85]. Infants [86] and people with diabetes may also be particularly at risk [85,87]. Generalized infections most often occur after surgery from burn wounds, leg ulcers, and pressure ulcer infections during diagnostic or therapeutic procedures in the urinary tract. Catheter-related infections, which can lead to meningitis, are reported especially in newborns and infants [88]. A dozen or *Enterococcus* species have been identified in human clinical samples. Among them, *E. faecalis* (80–90%) and *E. faecium* (5–15%) dominate, and these species are commonly associated with very serious complications and hospital infections [17].

A patient whose gastrointestinal tract is colonized by enterococci and undergoes diagnostic and therapeutic procedures during hospitalization, including antibiotic therapy, may be a source of drug-resistant *Enterococcus* isolates. The hospital is considered to be a reservoir of drug-resistant enterococci (e.g., high-level ampicillin resistance (Pbp5-R); high-level aminoglycoside resistance; glycopeptides resistance (vancomycin and teicoplanin); oxazolidinones resistance) [37,84,86,89,90]. Medical personnel and specifically the hands of health care workers are considered a vector for these resistant bacteria and are likely the main sources by which enterococci spread throughout the hospital [37]. It is mainly patients hospitalized in the vicinity of already colonized people who are at risk of exogenous infections due to multidrug-resistant enterococci [84]. *E. faecalis* and *E. faecium* are two of the major etiological factors of urinary tract infections, especially in people with structural abnormalities or following catheterization [91]. By colonizing catheters (long-term catheterization: >28 days) with hospital strains, bacteriuria with catheter-associated urinary tract infection symptoms may occur. *E. faecalis* likely acts as a pioneering species which, by infiltrating catheters, creates a medium for the colonization of other bacterium such as *P. mirabilis* [92,93]. Finally, studies have shown that the secretory factors of *E. faecalis* enhance the pathogenicity potential of *P. mirabilis* and, as a co-occurring bacteria, contribute to the destruction of tissues and bacteremia [93].

### 4.2. Bacterial Translocation from the GI Tract to Organs

Translocation from the gastrointestinal tract to various organs has been demonstrated in the enterococcal microbiota [44,94,95,96,97]. Both *E. faecalis* and *E. faecium* are invasive bacteria that pass through the intact mucosal epithelium and enter the host’s tissues. The bacteria translocate through the lamina propria mucosae to the mesenteric lymph node and from there to the circulatory system. In vitro studies on HT-29 and T84 cell lines infected with *Enterococcus* strains have shown bacterial factors that favor colonization and aggregation into the extracellular matrix, such as aggregatory substances, enterococcal polysaccharides, Epa antigen (responsible for the survival of bacteria in phagosomes), and gelatinase, promote the migration of bacteria [98,99]. In patients undergoing intensive chemotherapy or long-term broad-spectrum antibiotic therapy, the intestinal barrier and oral mucosa are damaged. Competition among enterococci within the intestinal microbiota may lead to the enrichment of those bacteria that better adapt to the ecological niche. Even strains considered to be probiotic may pose a threat to the patient when considering dominance within a sterilized medium (such as in the intestine).

Cases of enterococcal translocation to the lymph nodes, blood, liver, and spleen have been described [96,97]. Vieira et al. demonstrated that the so-called *E. gallinarum* strain can cause autoimmunity in genetically predisposed hosts [100]. *E. gallinarum*-specific DNA was recovered from liver biopsies of autoimmune patients. Such strains are referred to as pathobiontic (pathogenic bacteria originating from the microbiota). The translocation of bacteria from the gut into the blood stream has been reported in oncological and immunosuppressed patients, mainly those involving *E. coli* [41,42]. Cases of enterococcal sepsis or endocarditis [94,101,102,103] are less frequent and are the result of translocation from the gut [44,94,104]. In mice, Archambaud et al. [44] showed that the translocation of *E. faecalis* can occur across the intestinal mucosa and proved that the intestinal translocation of enterococci requires a threshold level of enterococcal hyperplasia in the intestinal lumen.

### 4.3. Mutagenic Effects and Theories of Tumorigenesis

Wang et al. [104] described that enterococci are also responsible for mutagenic effects. *E. faecalis* producing extracellular superoxide may induce chromosome breaking factors. Moreover, under experimental conditions on immortalized human and non-transformed murine colonic epithelial cells, *E. faecalis* can generate foci of aneuploidy, tetraploidy, and gamma-H2AX. In addition, the direct exposure of *E. faecalis* to these cells induced a G2 cell cycle arrest, hence the suggestion that commensals, including intestinal *E. faecalis*, may contribute to cellular transformation and tumorigenesis [104].

An association of *E. faecalis* translocation with colorectal carcinogenesis has been reported [105]. However, the role of enterococci in the development of colorectal cancer is still controversial [18]. Some authors suggest a protective role (e.g., *Enterococcus faecium* 137v (EF137v) [106], while others have indicated harmful effects. *E. faecalis* overgrowth usually occurs in the feces of colorectal cancer patients [107]. The involvement of *E. faecalis* in intestinal neoplasms can damage colonic epithelial cell DNA through the production of reactive oxygen and nitrogen species in the fermentation process [108]. Stimulation of macrophage activity [109] or changes in oxygen concentration can further activate oncogenes or inactivate tumor-suppressor genes [110,111]. The relationship between *E. faecalis* with various types of colorectal polyps thought to be a common cause of colorectal cancer have been documented as well [105]. Contrarily, in adenomatous polyposis coli mutant mice studies, the administration of a heat-killed strain of *E. faecalis* EC-12 reduced the development of polyps in the small intestine through the suppression of β-catenin signaling [105]. Grootaert et al. [112] demonstrated that *E. faecalis* grown on an aggressive colorectal cancer cell line (HCT-116) decreased the expression of FIAF protein similar to angiopoietin 4, which is typically detected in certain cancers.

### 4.4. Food-Borne Enterococci

The enterococci threat is not only observed in the hospital. The presence of enterococci in food is the result of contamination due to poor hygiene. *E. faecalis* and/or *E. faecium* are most often responsible for artisanal and traditional cheese contamination; however, other species have also been found (*E. casseliflavus*, *E. durans*, *E. hirae* and *E. gallinarum)* [113]. For example, in work by Gelsomino et al., *E. casseliflavus* and *E. faecalis* were isolated from food from a bulk-milk storage tank [12]. Poultry meat may also become contaminated with *E. faecalis* and *E. faecium* during processing and is frequently encountered [114]. The number of enterococci present in poultry meat range from 10^1^ to 10^3^ CFU/gr of raw chicken or turkey meat [15,115].

The widespread use of antimicrobials and intensive trade favors the emergence and spread of resistant microorganisms. The food chain is the key site where resistance is transmitted between the environment and humans. Moreover, a similarity in bacterial resistance profiles has been discovered between clinical material and food. Most frequently, these bacterial strains display resistance to streptomycin, erythromycin, tetracycline, and rifampicin [113]. The horizontal transfer of genes encoding resistance to aminoglycosides, tetracyclines, and macrolides in *Enterococcus* strains isolated from ready-to-eat dishes was documented by Chajęcka-Wierzchowska et al. [116]. The transfer of resistance to tetracyclines in enterococcal strains was also observed with a frequency ranging from 1.3 × 10^−6^ to 8.7 × 10^−7^ transconjugants/donor, for macrolides from 3.2 × 10^−6^ to 2.4 × 10^−8^ transconjugants/donor, and for genes encoding aminoglycosides from 1.7 × 10^−6^ to 3.2 × 10^−8^ transconjugants/donor. According to Haug et al., the high number of food-borne enterococci carrying resistance genes may significantly reduce the effectiveness of antibiotic therapy in intestinal infections [117,118]. Due to its highly adaptive capabilities, enterococci present in food are in a transient or permanent state to colonize the digestive tract, and this increases the danger of gene transfer to the intestinal microflora.

Enterococci can also cause food spoilage [119]. They produce thermostable amines such as tyramine, histamine, phenylethylalanine, cadaverine, and putrescine, which can cause allergic reactions or poisoning [15]. Problems are resistance to extremes used in food technology such as temperature as well as high pH and salinity.

## 5. Virulence Factors of *Enterococcus* spp. and Pathogenicity

*E. faecalis* and *E. faecium* strains are potentially pathogenic due to their special ability to adapt and survive in new environmental conditions [120]. These bacteria have developed mechanisms that facilitate and promote the colonization of biotic and abiotic surfaces and have the ability to evade the immune system [83], enabled by both the innate characteristics and the plasticity of their genome. Bacteria carry genes encoding virulence factors responsible for pathogenicity. Enterococci virulence factors can be grouped into several classes: (a) externally secreted, e.g., cytolysin, gelatinase, and serine protease; (b) surface proteins, e.g., Acm/Ace adhesins, Ebp pili, and extracellular surface protein Esp; (c) other virulence factors—hyaluronidase [121].

Many studies have confirmed that *E. faecium*, similar to *E. faecalis*, has the ability to bind collagen present on the surfaces of human cells. The microbial surface components recognizing adhesive matrix molecules (MSCRAMMs) [122] are responsible for this. Some of the best characterized MSCRAMMs molecules are Ace (a collagen-binding protein) for *E. faecalis* [122] and Acm for *E. faecium* [123]. These proteins bind to type I collagen and, to a lesser extent, to type IV collagen, and support the early colonization of various tissues. The participation of the Ace protein of *E. faecalis* has been described in the colonization of the heart valves and, consequently, in endocarditis [124]. The *acm* gene is most often detected in clinical, multi-drug resistant strains of *E. faecium* [125]; however, collagen binding proteins have also been found in isolates from healthy vectors.

The aggregation substances (AS) are responsible for the adhesion of enterococci to eukaryotic cells. The aggregation substances of bacterial cells are of plasmid-born origin. The best known conjugation plasmids containing the genes encoding these proteins are pPD1—Asp1 protein, pCF10—Asc10 protein, and pAD1—Asa1 protein [126,127,128]. Interestingly, AS proteins have been shown to be involved in the adhesion to and the penetration into intestinal cells, indicating that they may play a role in the translocation of *E. faecalis* through the intestinal wall [129].

Another virulence factor is the extracellular surface protein Esp. Esp is an adhesin occurring in various forms, allowing it to avoid the host’s immune defense mechanisms [130]. In addition, it participates in biofilm formation, which significantly increases the viability of bacteria in biopolymers (amyloid-like fibers) and may also be involved in antimicrobial resistance [131]. Biofilm production allows enterococci to avoid phagocytic attacks and makes it difficult to eradicate them. The multispecies biofilm environment is also conducive to the exchange of genes related to virulence. The *esp* gene, detected in both *E. faecalis* and *E. faecium*, occurs on pathogenicity islands (PAIs) and can be transmitted by conjugation [132].

Pili are encoded by the *ebp* operon (endocarditis- and biofilm-associated pili) and are involved in biofilm formation as well. Similar to adhesins, pili allow for the binding of collagen, fibrinogen, and thrombocytes [84]. In addition to *ebp*, the virulence genes *efa*Afs and *efa*Afm, which encode adhesion-like endocarditis antigens for *E. faecalis* and *E. faecium*, respectively, also contribute to endocarditis [133,134].

Cytolysin (Cyl-*β*-haemolysin) is a bacteriocin encoded on pheromone-responsive plasmids or is located on pathogenicity islands within the chromosome [130]. Cyl lyses red blood cells and some human white blood cells and is active against some Gram-positive bacteria [135].

The participation of gelatinase (GelE) [136,137] and serine protease (SprE) [138] in pathogenesis has also been observed. The main role of these proteins is to provide nutrients to bacteria by breaking down the host tissue and by participating in biofilm formation [139]. In addition, gelatinase is important for the translocation of *E. faecalis* across human enterocytes and facilitates microbial invasion [99].

The increased virulence of clinical strains is due to the presence of hyaluronidase, an enzyme that acts on hyaluronic acid and breaks down connective tissue through the depolymerization of mucopolysaccharide moieties [31,140]. In conjunction with toxin secretion, this enables *E. faecium* to more easily spread throughout a host’s tissues. The *hyl* gene encodes hyaluronidase, which is genetically programmed in megaplasmids and is present in many pathogenic enterococci.

Extracellular peroxides are an important factor for virulence and mainly occur in *E. faecalis* strains. They promote the survival of enterococci inside of the phagosome and damage the epithelium of the gastrointestinal tract, facilitating the exit of phagocytic cells from the intestine [108]. These strains are mainly isolated from patients with bacteremia [141]. A role has also been suggested for peroxidases in the formation of colorectal neoplasms [120].

The virulence factors are detected not only in clinical strains but also in bacterial strains from food. Genes encoding adhesion factors such as *esp*, *asa1*/*agg*, and *efaA* are highly prevalent among *E. faecalis* and *E. faecium* [142]. On the contrary, these genes are rarely reported in *E. durans* [143], *E. hirae* [144], and *E. casseliflavus* [145]. Finally, in food-borne strains, *cyl*, *gel*, and *hyl* are detected but with much lower frequency compared to clinic enterococci [143,146].

Another increasingly common feature of enterococci is the presence of pathogenicity islands, where the virulence genes involved in aggregation, cytolysin, or Esp as well as transcription factors regulating bile acid hydrolases are located. The formation and genetic instability of PAIs is the result of horizontal gene transfer (HGT), a process that is well-known for its contribution to microbial evolution. Many of the discussed genes encoding virulence factors (e.g., *as*, *cyl*, *hyl*) are also located on conjugation plasmids. HGT as a mechanism for genetic variation through gene acquisition in PIAs and the role of mobile genetic elements in the evolution of *E. faecalis* have been proven many times [147]. HGT is involved in spreading unfavorable and risk-raising features of enterococci, increasing the chances of these commensal bacteria to become pathogenic [148]. Moreover, HGT is responsible for the transfer of mobile genetic elements (e.g., plasmids) to other unrelated species [149]. Vignaroli et al. [150] observed the transfer of the *vanA* and *erm* (3) genes from porcine *E. faecium* and *E. durans* isolates to human *E. faecium*.

It should be noted that some determinants of enterococcal virulence are desirable in probiotic strains. This includes aggregation factors, exopolysaccharide (EPS) production, and the proteolytic system. Aggregation substances improve the likelihood of probiotic strain adherence to the host’s intestinal epithelium and are therefore an important feature for the efficient colonization of the GI tract along with indirectly affecting immunomodulation and providing protection against pathogens. The aggregation substances (ASs) on the cell surfaces of bacteria induce cell aggregation (and auto-aggregation) and are responsible for biofilm production. The enterococcal aggregation protein, AggE, is found in probiotic strains such as *Enterococcus faecium* BGGO9-28 and possesses high adhesive capabilities to collagen, fibronectin, and mucin [151]. The competitive formation of non-pathogenic biofilms promotes the elimination of harmful bacteria through pH alteration and competition for nutrients [151].

EPS is an exometabolite composed of β-1,6-linked poly-N-acetylglucosamine (polyGlcNAc)-containing polymers. The production of EPS can be considered a virulence trait [152]; however, EPS also facilitates the adhesion of probiotic enterococci through cooperation and the aggregation of cells [153]. The synthesis of EPS allows for the movement of this non-motile bacterium to an environment with nutrients and allows it to escape stressful conditions (higher pH, temperature, osmolarity), toxic conditions (e.g., antibiotics, metal ions, bile salts, gastric and pancreatic enzymes), or even evade the human immune response [154]. EPS can exert antagonistic activity against Gram-positive and Gram-negative pathogens, but the potential mechanisms are difficult to explain. It is suggested that EPS accumulates metabolites that adversely affect other bacteria [155], and these metabolites may also disrupt the structure of peptidoglycan and block receptors and channels on the outer membrane of the Gram-negative bacteria [156,157].

From a practical point of view, EPS-production is desirable because it improves the viscosity and texture of dairy products and can be used by the food industry to control biofilm-production by bacteria [142].

*Enterococcus strains* also display proteolytic activity (producing of extracellular proteinases, intracellular peptidases, and transport enzymes) [158,159] and play an important role in bacterial growth [158]. Some of them, including extracellular-secreted (E) or cell envelope proteases are used in the fermentation of dairy products) [142,160].

Bacteriocins constitute a functionally diverse family of toxins that are ribosomally synthesized peptides or proteins. Enterocins are used in dairy products, meat, fish, and plant-derived products (enterocin RM6, CRL35, AS-48) as beneficial additives in food production. Currently, bacteriocins are also being considered as promising candidates to treat infections caused by multi-drug resistant pathogens, e.g., in GI-tract diseases (enterocin A, S760, E50–52) and skin infections (enterocin A-48) [158]. In conclusion, some features of enterococci, such as virulence factors, make them an intermediate between emerging pathogens and potential probiotics.

## 6. The Problem of Antibiotic Resistance

The uncontrolled prophylactic use of antibiotics in a hospital environment and on animal farms has resulted in a gradual build-up of resistance among enterococci [126,161]. It has been proven that the commensal genome of enterococci can evolve greater pathogenicity through adaptation to the hospital environment [84]. Likewise, the European Center for Disease Prevention and Control has estimated that 37,000 people die due to infection caused by multidrug-resistant bacteria as a result of Hospital Acquired Infections every year [162].

Research indicates the development of ampicillin resistance and associated resistance to ciprofloxacin is the main phenotypic marker of hospital *E. faecium* isolates, a marker that precedes resistance to glycopeptides by several years [49,163,164]. In some countries, tetracycline is one of the most frequently used antibiotics for human and animal infections due to its availability and low cost [165]. However, the extensive use of tetracyclines has often led to the emergence of resistant bacteria [166]. In one hospital in Italy, a 2-year retrospective analysis of antimicrobial drug resistance and the spread of nosocomial infection found that about 70% of *E. faecalis* isolated from clinical patients had resistance to tetracycline and erythromycin [164]. The most commonly encountered tetracycline-resistant determinant in enterococci is tet(M), which is mainly associated with a conjugative transposon, particularly Tn916 [151].

Resistance to vancomycin (mainly VanA and VanB phenotype) is also becoming more frequent [84]. VanA resistance is characterized by a high degree of vancomycin- and teicoplanin-induced resistance. It is most often found in *E. faecium* strains, but it is also found in *E. faecalis* and, to a lesser extent, in *E. durans*, *E. raffinosus*, *E. hirae*, *E. avium*, and *E. gallinarum*. The genes determining this type of resistance are found on the Tn1546 transposon, which may be on a plasmid or may integrate with the bacterial chromosome [167,168]. The most important factor in the outbreak of hospital vancomycin-resistant enterococci is the colonization of the excretory system, which almost always precedes bacteremia and is the main reservoir from which the spread of microorganisms in the hospital environment takes place.

Among multidrug-resistant treatments, linezolid was once the drug of last resort. Resistance to linezolid (linezolid-resistant Enterococcus) has now been observed for several years against clinical isolates of the genus *Enterococcus*. New medicinal products have been introduced, such as dalbavancin (a lipopeptide), oritavancin and telavancin (glycopeptides), and tedizolide (oxazolidinone, the successor of linezolide). However, the activity of these drugs against enterococci and their availability in different countries varies considerably [169]. Therefore, drug-resistant enterococci infections pose a significant epidemiological and therapeutic problem.

Antibiotics are used not only for therapeutic and prophylactic purposes; they are also to protect consumers against microorganisms that may contaminate farms and animal products [168]. Enterococci are also pathogens of farm animals, and the abuse of antibiotics in veterinary medicine by animal breeders and by food producers will contribute to the deepening of this multi-drug resistance phenomenon. Resistance to ciprofloxacin, norfloxacin, tetracyclines, and even linezolid have been found in strains isolated from sausage, cheese, fish, and fish products [170]. In foods of animal origin produced in Europe, isolates resistant to gentamicin and streptomycin are rare, while in the United States, they are quite common [171]. Antibiotic resistance in the enterococcal strains commonly used as starter cultures for biotechnological applications in the dairy industry has also been identified. It is known that these strains must be sensitive to relevant clinical antibiotics. In a study by Terzić-Vidojević et al., [172], enterococci (with predominant species: *Enterococcus durans*, *Enterococcus faecalis*, and *Enterococcus faecium*) isolated from dairy products from different regions of the Western Balkan countries of Serbia, Croatia, Bosnia, and Herzegovina showed resistance to various antibiotics. They found that 185 out of 636 isolates were susceptible to tested antibiotics, and five of them met the criteria for the starter cultures (without any gene encoding virulence factors and in the absence of biogenic amines). A significant portion of the strains isolated from dairy products turned out to be useless due to drug resistance.

Eating raw and processed food contaminated with multi-drug resistant microorganisms can pose a threat to human life and health. Jahan et al. [173] demonstrated that the gene determining resistance to tetracycline and streptomycin was transferred from food-derived *E. faecium* and *E. faecalis* strains to clinical strains. The overuse of feed antibiotics in breeding has also made *Enterococcus* bacteria cross-resistant to vancomycin and teicoplanin. Cylactin has been proposed as an alternative to antibiotics along with the probiotic strain *E. faecium* NCIMB 10415, which protects piglets against diarrhea by competing with pathogenic strains of *E. coli* and *Salmonella* spp. [174].

Zoonotic transmission of drug-resistant enterococci (*E. faecium* and *E. faecalis*, and much less frequently *E. durans*, *E. casseliflavus* and *E. gallinarum*) from animals to humans through contact with animal secretions and excretions (dogs are a more common reservoir of drug-resistant enterococci than cats) [175] represents another issue. Studies conducted in various countries show a close relationship between vancomycin-resistant enterococcal species isolated from dogs with isolates responsible for nosocomial infections in humans [176].

The use of probiotic *Enterococcus* strains is controversial despite their beneficial effects in humans and animals. The reason for this is because of the bacterial acquisition of genes encoding resistance to glycopeptide antibiotics (vancomycin) and resistance to high concentrations of aminoglycosides (high level aminoglycoside resistance). Horizontal gene transfer from pathogenic enterococci to strains of commensals and other species of bacteria constituting the physiological microflora of the gastrointestinal tract has been reported [177]. In this context, it should be noted that the digestive tract is an excellent environment for bacterial growth and for the exchange of genetic material between microbes.

## 7. Conclusions

**Figure 2:**
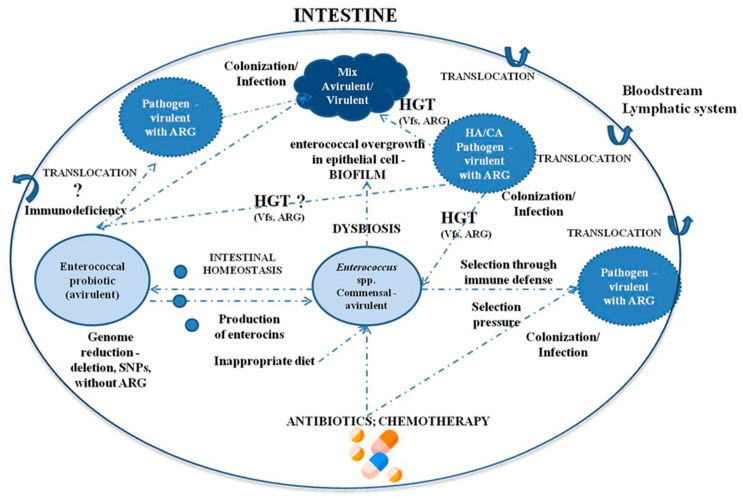
The relationships between enterococci in the transition towards pathogenicity. Legend: HGT—horizontal gene transfer; Vfs—virulence factors, ARG—antibiotic resistance gene. Antibiotics, chemotherapeutics, and diet can alter the composition of the gut microbiota. The overgrowth of *Enterococcus* causes dysbiosis and often disorders homeostasis. Biofilm is a key process in horizontal gene transfer (HGT). Transfer antibiotic resistance genes and virulence factors are facilitated. Commensal strains and probiotic strains can convert into pathogenic strains. For oncological patients and people with a weakened immune system, the translocation of pathogenic strains into the circulatory system is highly likely.

Enterococci represent one of the most controversial groups of bacteria. They are mainly commensal organisms isolated from humans, animals, plants, and insects. These bacteria affect the intestinal balance and modulate the human immune system. Due to their beneficial effects, selected strains are used as probiotics in numerous therapies and are also of biotechnological importance in the food industry. However, it should not be forgotten that in high-risk patients, enterococci may show potential for pathogenicity, especially in a hospital environment. *E. faecalis* and *E. faecium* display complex mechanisms of virulence that enable their colonization in various host tissues. The epidemic importance of *E. faecalis* and *E. faecium* is not new to clinicians, nor is the threat of increasing antibiotic resistance. We should remember that enterococcal overgrowth in the intestine and biofilm formation facilitates communication between bacteria and gene exchange through HGT. The use of selected probiotic enterococcal strains to support the treatment of patients may be an effective therapy; however, it should be remembered that enterococci have many faces. Due to their plastic genomes, enterococcal treatments should be used with caution, especially in immunodeficient patients. The probable relationships between enterococci of various phenotypes (probiotic, commensal, and pathogenic) in the transition towards pathogenicity are shown in Figure 2. The advantages and disadvantages of enterococci are compiled in Table 1.

Enterococci do not possess a Qualified Presumption of Safety status in the EU and are not generally regarded as safe in the USA. Hence, in order to ensure the safety of using *Enterococcus* as probiotics or starter cultures, further investigations on their genotypic and phenotypic characteristics need to be conducted before they are put into use. Currently, molecular biology techniques (e.g., PCR, whole genome sequencing) and classical susceptibility assays are used to detect virulence determinants and antibiotic resistance. In this way, producers can control enterococci for medical applications, as supplements, or in the food industry. In addition to detecting antibiotic resistance and virulence determinants, we should investigate useful features such as the hydrophobicity, auto-aggregation and co-aggregation ability, adhesion ability of strains to human intestinal cells, EPS production ability, antimicrobial activity, and the detection of genes encoding useful enterocins.

## Figures and Tables

**Table 1:** Advantages and disadvantages of enterococci.

**Advantages of Enterococci**	**Reference**
***Commensals***	
Immune homeostasis	[9,10]
Immunomodulatory effect	[10,44,45,46,63,64,65,78]
Producing bacteriocins against pathogens	[53,73,74,158]
Metabolism of carbohydrates and proteins—role in digestion	[14,15]
Blocking the spread of putrefactive bacteria	[14]
Lowering cholesterol levels	[63,66,75]
Protective role against cancer	[76]
***Probiotics***	
Biotherapeutic—e.g., chronic sinusitis, bronchitis	[13,50]
Bio-preservatives and hygiene indicator in food production	[12,14,15]
Dietary supplementation for animals	[46,48,67,69,70,77,78]
Starter cultures in dairy products	[11,14,15,80]
After treatment with antibiotics and as treatment for vancomycin-resistant enterococci colonization	[178]
**Disadvantages of Enterococci**	**Reference**
Potential pathogens (e.g., urinary tract infections, endocarditis)	[83,84,85,92,96,101,102,103,104,133,134]
translocation in the circulatory system (sepsis, bacteremia)	[41,44,94,95,96,97]
Nosocomial infection/hospital outbreak	[17,84,86,89,90]
Virulence and resistance factors can be transmitted between species or genera by horizontal gene transfer—a problem in hospital settings	[116,147,148,150,164,177]
Responsible for allergic reactions	[15]
Food spoilage	[15,115,119]
Food poisoning (foodborne pathogens)	[15]
Polyp formation and colorectal cancer	[18,108,110,111]

## Data Availability

Not applicable.

## References

[1] Sadowy E., Luczkiewicz A. (2014). Drug-resistant and hospital-associated Enterococcus faecium from wastewater, riverine estuary and anthropogenically impacted marine catchment basin. BMC Microbiol..

[2] Martin J.D., Mundt J.O. (1972). Enterococci in Insects. Appl. Microbiol..

[3] Nowakiewicz A., Ziółkowska G., Trościańczyk A., Zięba P., Gnat S. (2017). Determination of resistance and virulence genes in *Enterococcus faecalis* and *E. faecium* strains isolated from poultry and their genotypic characterization by ADSRRS-fingerprinting. Poult. Sci..

[4] Micallef S.A., Rosenberg Goldstein R.E., George A., Ewing L., Tall B.D., Boyer M.S., Joseph S.W., Sapkota A.R. (2013). Diversity, distribution and antibiotic resistance of *Enterococcus* spp. recovered from tomatoes, leaves, water and soil on U.S. Mid-Atlantic farms. Food Microbiol..

[5] Abriouel H., Omar N.B., Molinos A.C., López R.L., Grande M.J., Martínez-Viedma P., Ortega E., Cañamero M.M., Galvez A. (2008). Comparative analysis of genetic diversity and incidence of virulence factors and antibiotic resistance among enterococcal populations from raw fruit and vegetable foods, water and soil, and clinical samples. Int. J. Food Microbiol..

[6] Franz C.M.A.P., Schillinger U., Holzapfel W.H. (1996). Production and characterization of enterocin 900, a bacteriocin produced by Enterococcus faecium BFE 900 from black olives. Int. J. Food Microbiol..

[7] Mundt J.O. (1963). Occurrence of Enterococci on Plants in a Wild Environment. Appl. Microbiol..

[8] Müller T., Ulrich A., Ott E.M., Müller M. (2001). Identification of plant-associated enterococci. J. Appl. Microbiol..

[9] Saillant V., Lipuma D., Ostyn E., Joubert L., Boussac A., Guerin H., Brandelet G., Arnoux P., Lechardeur D. (2021). A novel enterococcus faecalis heme transport regulator (Fhtr) senses host heme to control its intracellular homeostasis. MBio.

[10] Laissue J.A., Chappuis B.B., Müller C., Reubi J.C., Gebbers J.O. (1993). The intestinal immune system and its relation to disease. Dig. Dis..

[11] García-Díez J., Saraiva C. (2021). Use of starter cultures in foods from animal origin to improve their safety. Int. J. Environ. Res. Public Health.

[12] Gelsomino R., Vancanneyt M., Condon S., Swings J., Cogan T.M. (2001). Enterococcal diversity in the environment of an Irish Cheddar-type cheesemaking factory. Int. J. Food Microbiol..

[13] Franz C.M.A.P., Huch M., Abriouel H., Holzapfel W., Gálvez A. (2011). Enterococci as probiotics and their implications in food safety. Int. J. Food Microbiol..

[14] Giraffa G. (2003). Functionality of enterococci in dairy products. Int. J. Food Microbiol..

[15] Giraffa G. (2002). Enterococci from foods. FEMS Microbiol. Rev..

[16] O’Driscoll T., Crank C.W. (2015). Vancomycin-resistant enterococcal infections: Epidemiology, clinical manifestations, and optimal management. Infect. Drug Resist..

[17] Guzman Prieto A.M., van Schaik W., Rogers M.R.C., Coque T.M., Baquero F., Corander J., Willems R.J.L. (2016). Global emergence and dissemination of enterococci as nosocomial pathogens: Attack of the clones?. Front. Microbiol..

[18] Cheng Y., Ling Z., Li L. (2020). The Intestinal Microbiota and Colorectal Cancer. Front. Immunol..

[19] Thomas A.M., Segata N. (2019). Multiple levels of the unknown in microbiome research. BMC Biol..

[20] Yang X., Xie L., Li Y., Wei C. (2009). More than 9,000,000 unique genes in human gut bacterial community: Estimating gene numbers inside a human body. PLoS ONE.

[21] Li J., Jia H., Cai X., Zhong H., Feng Q., Sunagawa S., Arumugam M., Kultima J.R., Prifti E., Nielsen T. (2014). An integrated catalog of reference genes in the human gut microbiome. Nat. Biotechnol..

[22] Qin J., Li R., Raes J., Arumugam M., Burgdorf K.S., Manichanh C., Nielsen T., Pons N., Levenez F., Yamada T. (2010). A human gut microbial gene catalogue established by metagenomic sequencing. Nature.

[23] De Filippo C., Cavalieri D., Di Paola M., Ramazzotti M., Poullet J.B., Massart S., Collini S., Pieraccini G., Lionetti P. (2010). Impact of diet in shaping gut microbiota revealed by a comparative study in children from Europe and rural Africa. Proc. Natl. Acad. Sci. USA.

[24] Koenig J.E., Spor A., Scalfone N., Fricker A.D., Stombaugh J., Knight R., Angenent L.T., Ley R.E. (2011). Succession of microbial consortia in the developing infant gut microbiome. Proc. Natl. Acad. Sci. USA.

[25] Arumugam M., Raes J., Pelletier E., Le Paslier D., Yamada T., Mende D.R., Fernandes G.R., Tap J., Bruls T., Batto J.M. (2011). Enterotypes of the human gut microbiome. Nature.

[26] Kurokawa K., Itoh T., Kuwahara T., Oshima K., Toh H., Toyoda A., Takami H., Morita H., Sharma V.K., Srivastava T.P. (2007). Comparative metagenomics revealed commonly enriched gene sets in human gut microbiomes. DNA Res..

[27] Tap J., Mondot S., Levenez F., Pelletier E., Caron C., Furet J.P., Ugarte E., Muñoz-Tamayo R., Paslier D.L.E., Nalin R. (2009). Towards the human intestinal microbiota phylogenetic core. Environ. Microbiol..

[28] Murray B.E. (1990). The life and times of the enterococcus. Clin. Microbiol. Rev..

[29] Chenoweth C., Schaberg D. (1990). The epidemiology of enterococci. Eur. J. Clin. Microbiol. Infect. Dis..

[30] Gilmore M., Clewell D., Courvalin P., Dunny G., Murray B.E., Rice L.B. (2002). The Enterococci: Pathogenesis, Molecular Biology, and Antibiotic Resistance.

[31] Wan L.Y.M., Chen Z.J., Shah N.P., El-Nezami H. (2016). Modulation of Intestinal Epithelial Defense Responses by Probiotic Bacteria. Crit. Rev. Food Sci. Nutr..

[32] Mu C., Yang Y., Zhu W. (2016). Gut microbiota: The brain peacekeeper. Front. Microbiol..

[33] Gewolb I.H., Schwalbe R.S., Taciak V.L., Harrison T.S., Panigrahi P. (1999). Stool microflora in extremely low birthweight infants. Arch. Dis. Child. Fetal Neonatal Ed..

[34] Růžičková M., Vítězová M., Kushkevych I. (2020). The characterization of Enterococcus genus: Resistance mechanisms and inflammatory bowel disease. Open Med..

[35] Fisher K., Phillips C. (2009). The ecology, epidemiology and virulence of Enterococcus. Microbiology.

[36] McHugh C.P., Zhang P., Michalek S., Eleazer P.D. (2004). pH required to kill *Enterococcus faecalis* in vitro. J. Endod..

[37] Kramer A., Schwebke I., Kampf G. (2006). How long do nosocomial pathogens persist on inanimate surfaces? A systematic review. BMC Infect. Dis..

[38] Valdes A.M., Walter J., Segal E., Spector T.D. (2018). Role of the gut microbiota in nutrition and health. BMJ.

[39] Silva N., Igrejas G., Gonçalves A., Poeta P. (2012). Commensal gut bacteria: Distribution of Enterococcus species and prevalence of Escherichia coli phylogenetic groups in animals and humans in Portugal. Ann. Microbiol..

[40] Krawczyk B., Lewandowski K., Bronk M., Samet A., Myjak P.S., Kur J. (2003). Evaluation of a novel method based on amplification of DNA fragments surrounding rare restriction sites (ADSRRS fingerprinting) for typing strains of vancomycin-resistant Enterococcus faecium. J. Microbiol. Methods.

[41] Szemiako K., Krawczyk B., Samet A., Śledzińska A., Nowicki B., Nowicki S., Kur J. (2013). A subset of two adherence systems, acute pro-inflammatory pap genes and invasion coding dra, fim, or sfa, increases the risk of Escherichia coli translocation to the bloodstream. Eur. J. Clin. Microbiol. Infect. Dis..

[42] Samet A., Śledzińska A., Krawczyk B., Hellmann A., Nowicki S., Kur J., Nowicki B. (2013). Leukemia and risk of recurrent Escherichia coli bacteremia: Genotyping implicates E. Coli translocation from the colon to the bloodstream. Eur. J. Clin. Microbiol. Infect. Dis..

[43] Gibson G.R., Hutkins R., Sanders M.E., Prescott S.L., Reimer R.A., Salminen S.J., Scott K., Stanton C., Swanson K.S., Cani P.D. (2017). Expert consensus document: The International Scientific Association for Probiotics and Prebiotics (ISAPP) consensus statement on the definition and scope of prebiotics. Nat. Rev. Gastroenterol. Hepatol..

[44] Archambaud C., Derré-Bobillot A., Lapaque N., Rigottier-Gois L., Serror P. (2019). Intestinal translocation of enterococci requires a threshold level of enterococcal overgrowth in the lumen. Sci. Rep..

[45] CPG Sec. 689.100 Direct-Fed Microbial Products|FDA. https://www.fda.gov/regulatory-information/search-fda-guidance-documents/cpg-sec-689100-direct-fed-microbial-products.

[46] Bednorz C., Guenther S., Oelgeschläger K., Kinnemann B., Pieper R., Hartmann S., Tedin K., Semmler T., Neumann K., Schierack P. (2013). Feeding the probiotic Enterococcus faecium strain NCIMB 10415 to piglets specifically reduces the number of Escherichia coli pathotypes that adhere to the gut mucosa. Appl. Environ. Microbiol..

[47] Institute of Medicine (2006). Ending the War Metaphor. The Changing Agenda for Unraveling the Host-Microbe Relationship: Workshop Summary.

[48] Koutsoumanis K., Allende A., Alvarez-Ordóñez A., Bolton D., Bover-Cid S., Chemaly M., Davies R., De Cesare A., Hilbert F., Lindqvist R. (2020). Update of the list of QPS-recommended biological agents intentionally added to food or feed as notified to EFSA 11: Suitability of taxonomic units notified to EFSA until September 2019. EFSA J..

[49] Rusch K., Rusch V. (2001). Mikrobiologische Therapie Grundlagen und Praxis.

[50] Habermann W., Zimmermann K., Skarabis H., Kunze R., Rusch V. (2002). Reduction of acute relapses in patients with chronic recurrent hypertrophic sinusitis during treatment with a bacterial immunostimulant (*Enterococcus faecalis* bacteriae of human origin—A medical probiotic). Arzneimittel-Forschung/Drug Res..

[51] Zhou Y., Liang Y., Lynch K.H., Dennis J.J., Wishart D.S. (2011). PHAST: A Fast Phage Search Tool. Nucleic Acids Res..

[52] Fritzenwanker M., Kuenne C., Billion A., Hain T., Zimmermann K., Goesmann A., Chakraborty T., Domann E. (2013). Complete genome sequence of the probiotic *Enterococcus faecalis* Symbioflor 1 clone DSM 16431. Genome Announc..

[53] Wassenaar T.M., Marzorati2 M., Beimfohr C., Siegl A., Zimmermann K. (2017). Survival of Probiotic *E. coli* and *Ent. faecalis* in the Human Host after Oral Intake: Results from in Vitro and in Vivo Studies. Adv. Biotechnol. Microbiol..

[54] Domann E., Hain T., Ghai R., Billion A., Kuenne C., Zimmermann K., Chakraborty T. (2007). Comparative genomic analysis for the presence of potential enterococcal virulence factors in the probiotic *Enterococcus faecalis* strain Symbioflor 1. Int. J. Med. Microbiol..

[55] Baccouri O., Boukerb A.M., Farhat L.B., Zébré A., Zimmermann K., Domann E., Cambronel M., Barreau M., Maillot O., Rincé I. (2019). Probiotic Potential and Safety Evaluation of *Enterococcus faecalis* OB14 and OB15, Isolated from Traditional Tunisian Testouri Cheese and Rigouta, Using Physiological and Genomic Analysis. Front. Microbiol..

[56] Vebø H.C., Solheim M., Snipen L., Nes I.F., Brede D.A. (2010). Comparative genomic analysis of pathogenic and probiotic *Enterococcus faecalis* isolates, and their transcriptional responses to growth in human urine. PLoS ONE.

[57] Nami Y., Haghshenas B., Haghshenas M., Abdullah N., Khosroushahi A.Y. (2015). The Prophylactic effect of probiotic Enterococcus lactis IW5 against different human cancer cells. Front. Microbiol..

[58] Adnan M., Patel M., Hadi S. (2017). Functional and health promoting inherent attributes of Enterococcus hirae F2 as a novel probiotic isolated from the digestive tract of the freshwater fish Catla catla. PeerJ.

[59] Li B., Evivie S.E., Jin D., Meng Y., Li N., Yan F., Huo G., Liu F. (2018). Complete genome sequence of Enterococcus durans KLDS6.0933, a potential probiotic strain with high cholesterol removal ability. Gut Pathog..

[60] Wopereis H., Oozeer R., Knipping K., Belzer C., Knol J. (2014). The first thousand days—Intestinal microbiology of early life: Establishing a symbiosis. Pediatr. Allergy Immunol..

[61] Ouwehand A.C., Salminen S., Isolauri E. (2002). Probiotics: An overview of beneficial effects. Antonie Leeuwenhoek.

[62] Marteau P., Seksik P., Lepage P., Dore J. (2012). Cellular and Physiological Effects of Probiotics and Prebiotics. Mini-Rev. Med. Chem..

[63] Hlivak P., Odraska J., Ferencik M., Ebringer L., Jahnova E., Mikes Z. (2005). One-year application of probiotic strain Enterococcus faecium M-74 decreases serum cholesterol levels. Bratisl. Lek. Listy.

[64] Mikeš Z., Ferenčík M., Jahnová E., Ebringer L., Čižnár I. (1995). Hypocholesterolemic and immunostimulatory effects of orally applied Enterococcus fœcium M-74 in man. Folia Microbiol..

[65] Ebringer L., Ferenčík M., Lahitová N., Kačáni L., Michálková D. (1995). Anti-mutagenic and immuno-stimulatory properties of lactic acid bacteria. World J. Microbiol. Biotechnol..

[66] Mego M., Koncekova R., Mikuskova E., Drgona L., Ebringer L., Demitrovicova L., Nemova I., Trupl J., Mardiak J., Koza I. (2006). Prevention of febrile neutropenia in cancer patients by probiotic strain Enterococcus faecium M-74. Phase II study. Support. Care Cancer.

[67] European Food Safety Authority (EFSA) (2005). Opinion of the Scientific Panel on additives and products or substances used in animal feed (FEEDAP) on the updating of the criteria used in the assessment of bacteria for resistance to antibiotics of human or veterinary importance. EFSA J..

[68] Mitra A.K., Rabbani G.H. (1990). A double-blind, controlled trial of bioflorin (*Streptococcus faecium* SF68) in adults with acute diarrhea due to Vibrio cholerae and enterotoxigenic *Escherichia coli*. Gastroenterology.

[69] Bybee S.N., Scorza A.V., Lappin M.R. (2011). Effect of the probiotic Enterococcus faecium SF68 on presence of diarrhea in cats and dogs housed in an animal shelter. J. Vet. Intern. Med..

[70] Torres-Henderson C., Summers S., Suchodolski J., Lappin M.R. (2017). Effect of Enterococcus Faecium Strain SF68 on Gastrointestinal Signs and Fecal Microbiome in Cats Administered Amoxicillin-Clavulanate. Top. Companion Anim. Med..

[71] Al-Balawi M., Morsy F.M. (2020). *Enterococcus faecalis* Is a Better Competitor Than Other Lactic Acid Bacteria in the Initial Colonization of Colon of Healthy Newborn Babies at First Week of Their Life. Front. Microbiol..

[72] Giraffa G. (2014). Enterococcus. Encyclopedia of Food Microbiology II Enterococcus.

[73] Barrangou R., Lahtinen S.J., Ibrahim F., Ouwehand A.C., Lahtinen S., Ouwehand A.C., Salminen S., von Wright A. (2012). Chapter 5: Genus Lactobacillus. Lactic Acid Bacteria: Microbiological and Functional Aspects.

[74] Abanoz H.S., Kunduhoglu B. (2018). Antimicrobial activity of a bacteriocin produced by enterococcus faecalis kt11 against some pathogens and antibiotic-resistant Bacteria. Korean J. Food Sci. Anim. Resour..

[75] Wu Y., Zhen W., Geng Y., Wang Z., Guo Y. (2019). Effects of dietary Enterococcus faecium NCIMB 11181 supplementation on growth performance and cellular and humoral immune responses in broiler chickens. Poult. Sci..

[76] Viaud S., Saccheri F., Mignot G., Yamazaki T., Daillère R., Hannani D., Enot D.P., Pfirschke C., Engblom C., Pittet M.J. (2013). The intestinal microbiota modulates the anticancer immune effects of cyclophosphamide. Science.

[77] Benyacoub J., Czarnecki-Maulden G.L., Cavadini C., Sauthier T., Anderson R.E., Schiffrin E.J., Von der Weid T. (2003). von Supplementation of food with Enterococcus faecium (SF68) stimulates immune functions in young dogs. J. Nutr..

[78] Vahjen W., Taras D., Simon O. (2007). Effect of the probiotic Enterococcus faecium NCIMB10415 on cell numbers of total *Enterococcus* spp., *E. faecium* and *E. faecalis* in the intestine of piglets. Curr. Issues Intest. Microbiol..

[79] Lucena-Padrós H., González J.M., Caballero-Guerrero B., Ruiz-Barba J.L., Maldonado-Barragán A. (2014). Enterococcus olivae sp. nov., isolated from Spanish-style green-olive fermentations. Int. J. Syst. Evol. Microbiol..

[80] Bhardwaj A., Malik R.K., Chauhan P. (2008). Functional and safety aspects of enterococci in dairy foods. Indian J. Microbiol..

[81] Lebreton F., Willems R.J.L., Gilmore M.S. (2014). Enterococcus Diversity, Origins in Nature, and Gut Colonization. Enterococci: From Commensals to Leading Causes of Drug Resistant Infection [Internet].

[82] John U.V., Carvalho J. (2011). Enterococcus: Review of its physiology, pathogenesis, diseases and the challenges it poses for clinical microbiology. Front. Biol..

[83] Kao P.H.N., Kline K.A. (2019). Dr. Jekyll and Mr. Hide: How *Enterococcus faecalis* Subverts the Host Immune Response to Cause Infection. J. Mol. Biol..

[84] Krawczyk B., Wysocka M., Kotłowski R., Bronk M., Michalik M., Samet A. (2020). Linezolid-resistant Enterococcus faecium strains isolated from one hospital in Poland-commensals or hospital-adapted pathogens?. PLoS ONE.

[85] Tendolkar P.M., Baghdayan A.S., Shankar N. (2003). Pathogenic enterococci: New developments in the 21st century. Cell. Mol. Life Sci..

[86] Krawczyk B., Samet A., Bronk M., Hellmann A., Kur J. (2004). Emerging linezolid-resistant, vancomycin resistant Enterococcus faecium from a patient of a haematological unit in Poland. Pol. J. Microbiol..

[87] Fanaro S., Chierici R., Guerrini P., Vigi V. (2003). Intestinal microflora in early infancy: Composition and development. Acta Paediatr. Int. J. Paediatr. Suppl..

[88] Bretón J.R., Peset V., Morcillo F., Cano J., Sarrión A., Pérez-Belles C., Gobernado M. (2002). Neonatal meningitis due to *Enterococcus* spp.: Presentation of four cases. Enferm. Infecc. Microbiol. Clin..

[89] Montealegre M.C., Roh J.H., Rae M., Davlieva M.G., Singh K.V., Shamoo Y., Murray B.E. (2017). Differential Penicillin-Binding Protein 5 (PBP5) levels in the enterococcus faecium clades with different levels of ampicillin resistance. Antimicrob. Agents Chemother..

[90] Correa-Martinez C.L., Tönnies H., Froböse N.J., Mellmann A., Kampmeier S. (2020). Transmission of vancomycin-resistant enterococci in the hospital setting: Uncovering the patient–environment interplay. Microorganisms.

[91] O’Brien V.P., Hannan T.J., Nielsen H.V., Hultgren S.J. (2016). Drug and Vaccine Development for the Treatment and Prevention of Urinary Tract Infections. Microbiol. Spectr..

[92] Armbruster C.E., Prenovost K., Mobley H.L.T., Mody L. (2017). How Often Do Clinically Diagnosed Catheter-Associated Urinary Tract Infections in Nursing Homes Meet Standardized Criteria?. J. Am. Geriatr. Soc..

[93] Learman B.S., Brauer A.L., Eaton K.A., Armbruster C.E. (2020). A rare opportunist, *Morganella morganii*, decreases severity of polymicrobial catheter-associated urinary tract infection. Infect. Immun..

[94] Wells C.L., Jechorek R.P., Gillingham K.J. (1991). Relative Contributions of Host and Microbial Factors in Bacterial Translocation. Arch. Surg..

[95] Fine R.L., Vieira S.M., Gilmore M.S., Kriegel M.A. (2020). Mechanisms and consequences of gut commensal translocation in chronic diseases. Gut Microbes.

[96] Knoop K.A., McDonald K.G., Kulkarni D.H., Newberry R.D. (2016). Antibiotics promote inflammation through the translocation of native commensal colonic bacteria. Gut.

[97] Diehl G.E., Longman R.S., Zhang J.X., Breart B., Galan C., Cuesta A., Schwab S.R., Littman D.R. (2013). Microbiota restricts trafficking of bacteria to mesenteric lymph nodes by CX 3 CR1 hi cells. Nature.

[98] Zeng J., Teng F., Weinstock G.M., Murray B.E. (2004). Translocation of *Enterococcus faecalis* Strains across a Monolayer of Polarized Human Enterocyte-Like T84 Cells. J. Clin. Microbiol..

[99] Zeng J., Teng F., Murray B.E. (2005). Gelatinase is important for translocation of *Enterococcus faecalis* across polarized human enterocyte-like T84 cells. Infect. Immun..

[100] Manfredo Vieira S., Hiltensperger M., Kumar V., Zegarra-Ruiz D., Dehner C., Khan N., Costa F.R.C., Tiniakou E., Greiling T., Ruff W. (2018). Translocation of a gut pathobiont drives autoimmunity in mice and humans. Science.

[101] Eccles L.J., O’Neill P., Lomax M.E. (2011). Delayed repair of radiation induced clustered DNA damage: Friend or foe?. Mutat. Res.—Fundam. Mol. Mech. Mutagen..

[102] Escolà-Vergé L., Peghin M., Givone F., Pérez-Rodríguez M.T., Suárez-Varela M., Meije Y., Abelenda G., Almirante B., Fernández-Hidalgo N. (2020). Prevalence of colorectal disease in *Enterococcus faecalis* infective endocarditis: Results of an observational multicenter study. Rev. Esp. Cardiol. (Engl. Ed.).

[103] Frickmann H., Köller K., Veil I., Weise M., Ludyga A., Schwarz N.G., Warnke P., Podbielski A. (2017). On the role of enterococci in the bloodstream: Results of a single-center, retrospective, observational study at a German University Hospital. Eur. J. Microbiol. Immunol..

[104] Wang X., Allen T.D., May R.J., Lightfoot S., Houchen C.W., Huycke M.M. (2008). *Enterococcus faecalis* induces aneuploidy and tetraploidy in colonic epithelial cells through a bystander effect. Cancer Res..

[105] Rezasoltani S., Asadzadeh Aghdaei H., Dabiri H., Akhavan Sepahi A., Modarressi M.H., Nazemalhosseini Mojarad E. (2018). The association between fecal microbiota and different types of colorectal polyp as precursors of colorectal cancer. Microb. Pathog..

[106] de Andrade Calaça P.R., da Silva Santos D., da Silva J.F., Aragão A.B.L., de Melo I.M.F., da Silva E.C.S., Porto A.L.F., Soares M.T.C.V. (2021). Enterococcus faecium 137v como fator de proteção em modelo animal para câncer colorretal. Res. Soc. Dev..

[107] Wang T., Cai G., Qiu Y., Fei N., Zhang M., Pang X., Jia W., Cai S., Zhao L. (2012). Structural segregation of gut microbiota between colorectal cancer patients and healthy volunteers. ISME J..

[108] Huycke M.M., Abrams V., Moore D.R. (2002). *Enterococcus faecalis* produces extracellular superoxide and hydrogen peroxide that damages colonic epithelial cell DNA. Carcinogenesis.

[109] Allen T.D., Moore D.R., Wang X., Casu V., May R., Lerner M.R., Houchen C., Brackett D.J., Huycke M.M. (2008). Dichotomous metabolism of *Enterococcus faecalis* induced by haematin starvation modulates colonic gene expression. J. Med. Microbiol..

[110] Evans M.D., Dizdaroglu M., Cooke M.S. (2004). Oxidative DNA damage and disease: Induction, repair and significance. Mutat. Res.—Rev. Mutat. Res..

[111] Huycke M.M., Moore D.R. (2002). In vivo production of hydroxyl radical by *Enterococcus faecalis* colonizing the intestinal tract using aromatic hydroxylation. Free Radic. Biol. Med..

[112] Grootaert C., Van de Wiele T., Van Roosbroeck I., Possemiers S., Vercoutter-Edouart A.S., Verstraete W., Bracke M., Vanhoecke B. (2011). Bacterial monocultures, propionate, butyrate and H2O2 modulate the expression, secretion and structure of the fasting-induced adipose factor in gut epithelial cell lines. Environ. Microbiol..

[113] Fracalanzza S.A.P., Scheidegger E.M.D., Dos Santos P.F., Leite P.C., Teixeira L.M. (2007). Antimicrobial resistance profiles of enterococci isolated from poultry meat and pasteurized milk in Rio de Janeiro, Brazil. Mem. Inst. Oswaldo Cruz.

[114] Bortolaia V., Espinosa-Gongora C., Guardabassi L. (2016). Human health risks associated with antimicrobial-resistant enterococci and Staphylococcus aureus on poultry meat. Clin. Microbiol. Infect..

[115] Miranda J.M., Guarddon M., Mondragón A., Vázquez B.I., Fente C.A., Cepeda A., Franco C.M. (2007). Antimicrobial resistance in *Enterococcus* spp. strains isolated from organic chicken, conventional chicken, and turkey meat: A comparative survey. J. Food Prot..

[116] Chajęcka-Wierzchowska W., Zadernowska A., Zarzecka U., Zakrzewski A., Gajewska J. (2019). Enterococci from ready-to-eat food—Horizontal gene transfer of antibiotic resistance genes and genotypic characterization by PCR melting profile. J. Sci. Food Agric..

[117] Choi J.M., Woo G.J. (2015). Transfer of Tetracycline Resistance Genes with Aggregation Substance in Food-Borne *Enterococcus faecalis*. Curr. Microbiol..

[118] Haug M.C., Tanner S.A., Lacroix C., Stevens M.J.A., Meile L. (2011). Monitoring horizontal antibiotic resistance gene transfer in a colonic fermentation model. FEMS Microbiol. Ecol..

[119] Hugas M., Garriga M., Aymerich M.T. (2004). Functionalty of enterococci in meat products. Int. J. Food Microbiol..

[120] Gaca A.O., Lemos J.A. (2019). Adaptation to Adversity: The Intermingling of Stress Tolerance and Pathogenesis in Enterococci. Microbiol. Mol. Biol. Rev..

[121] Gilmore M.S., Coburn P.S., Nallapareddy S.R., Murray B.E. (2014). Enterococcal Virulence. Enterococci.

[122] Rich R.L., Kreikemeyer B., Owens R.T., LaBrenz S., Narayana S.V.L., Weinstock G.M., Murray B.E., Höök M. (1999). Ace is a collagen-binding MSCRAMM from *Enterococcus faecalis*. J. Biol. Chem..

[123] Nallapareddy S.R., Weinstock G.M., Murray B.E. (2003). Clinical isolates of Enterococcus faecium exhibit strain-specific collagen binding mediated by Acm, a new member of the MSCRAMM family. Mol. Microbiol..

[124] de Freitas Silva E.C., Montalvão C.R., Bonafé S. (2017). Infectious Endocarditis from *Enterococcus faecalis* Associated with Tubular Adenoma of the Sigmoid Colon. Case Rep. Infect. Dis..

[125] Nallapareddy S.R., Singh K.V., Okhuysen P.C., Murray B.E. (2008). A functional collagen adhesin gene, acm, in clinical isolates of Enterococcus faecium correlates with the recent success of this emerging nosocomial pathogen. Infect. Immun..

[126] Torabinejad M., Eby W.C., Naidorf I.J. (1985). Inflammatory and immunological aspects of the pathogenesis of human periapical lesions. J. Endod..

[127] Galli D., Friesenegger A., Wirth R. (1992). Transcriptional control of sex-pheromone-inducible genes on plasmid pAD1 of *Enterococcus faecalis* and sequence analysis of a third structural gene for (pPD1-encoded) aggregation substance. Mol. Microbiol..

[128] Kao S.M., Olmsted S.B., Viksnins A.S., Gallo J.C., Dunny G.M. (1991). Molecular and genetic analysis of a region of plasmid pCF10 containing positive control genes and structural genes encoding surface proteins involved in pheromone-inducible conjugation in *Enterococcus faecalis*. J. Bacteriol..

[129] Hendrickx A.P.A., Willems R.J.L., Bonten M.J.M., van Schaik W. (2009). LPxTG surface proteins of enterococci. Trends Microbiol..

[130] Shankar V., Baghdayan A.S., Huycke M.M., Lindahl G., Gilmore M.S. (1999). Infection-derived *Enterococcus faecalis* strains are enriched in esp, a gene encoding a novel surface protein. Infect. Immun..

[131] Mohamed J.A., Huang D.B. (2007). Biofilm formation by enterococci. J. Med. Microbiol..

[132] van Schaik W., Top J., Riley D.R., Boekhorst J., Vrijenhoek J.E.P., Schapendonk C.M.E., Hendrickx A.P.A., Nijman I.J., Bonten M.J.M., Tettelin H. (2010). Pyrosequencing-based comparative genome analysis of the nosocomial pathogen Enterococcus faecium and identification of a large transferable pathogenicity island. BMC Genom..

[133] Low Y.L., Jakubovics N.S., Flatman J.C., Jenkinson H.F., Smith A.W. (2003). Manganese-dependent regulation of the endocarditis-associated virulence factor EfaA of Enterococcus faecafis. J. Med. Microbiol..

[134] Nallapareddy S.R., Singh K.V., Sillanpää J., Garsin D.A., Höök M., Erlandsen S.L., Murray B.E. (2006). Endocarditis and biofilm-associated pili of *Enterococcus faecalis*. J. Clin. Investig..

[135] Mundy L.M., Sahm D.F., Gilmore M. (2000). Relationships between Enterococcal Virulence and Antimicrobial Resistance. Clin. Microbiol. Rev..

[136] Del Papa M.F., Hancock L.E., Thomas V.C., Perego M. (2007). Full activation of *Enterococcus faecalis* gelatinase by a C-terminal proteolytic cleavage. J. Bacteriol..

[137] Thurlow L.R., Thomas V.C., Narayanan S., Olson S., Fleming S.D., Hancock L.E. (2010). Gelatinase contributes to the pathogenesis of endocarditis caused by *Enterococcus faecalis*. Infect. Immun..

[138] Engelbert M., Mylonakis E., Ausubel F.M., Calderwood S.B., Gilmore M.S. (2004). Contribution of gelatinase, serine protease, and fsr to the pathogenesis of *Enterococcus faecalis* endophthalmitis. Infect. Immun..

[139] Ali L., Goraya M.U., Arafat Y., Ajmal M., Chen J.L., Yu D. (2017). Molecular mechanism of quorum-sensing in *Enterococcus faecalis*: Its role in virulence and therapeutic approaches. Int. J. Mol. Sci..

[140] Kayaoglu G., Ørstavik D. (2004). Virulence factors of *Enterococcus faecalis*: Relationship to endodontic disease. Crit. Rev. Oral Biol. Med..

[141] Huycke M.M., Spiegel C.A., Gilmore M.S. (1991). Bacteremia caused by hemolytic, high-level gentamicin-resistant *Enterococcus faecalis*. Antimicrob. Agents Chemother..

[142] Dapkevicius M.d.L.E., Sgardioli B., Câmara S.P.A., Poeta P., Malcata F.X. (2021). Current trends of enterococci in dairy products: A comprehensive review of their multiple roles. Foods.

[143] İspirli H., Demirbaş F., Dertli E. (2017). Characterization of functional properties of *Enterococcus* spp. isolated from Turkish white cheese. LWT—Food Sci. Technol..

[144] Nieto-Arribas P., Seseña S., Poveda J.M., Chicón R., Cabezas L., Palop L. (2011). Enterococcus populations in artisanal Manchego cheese: Biodiversity, technological and safety aspects. Food Microbiol..

[145] Fuka M.M., Maksimovic A.Z., Tanuwidjaja I., Hulak N., Schloter M. (2017). Characterization of enterococcal community isolated from an Artisan Istrian raw milk cheese: Biotechnologicaland safety aspects. Food Technol. Biotechnol..

[146] Özkan E.R., Demirci T., Akın N. (2021). In vitro assessment of probiotic and virulence potential of Enterococcus faecium strains derived from artisanal goatskin casing Tulum cheeses produced in central Taurus Mountains of Turkey. LWT.

[147] McBride S.M., Coburn P.S., Baghdayan A.S., Willems R.J.L., Grande M.J., Shankar N., Gilmore M.S. (2009). Genetic variation and evolution of the pathogenicity island of *Enterococcus faecalis*. J. Bacteriol..

[148] Hacker J., Kaper J.B. (2000). Pathogenicity islands and the evolution of microbes. Annu. Rev. Microbiol..

[149] Sparo M., Urbizu L., Solana M.V., Pourcel G., Delpech G., Confalonieri A., Ceci M., Sánchez Bruni S.F. (2012). High-level resistance to gentamicin: Genetic transfer between *Enterococcus faecalis* isolated from food of animal origin and human microbiota. Lett. Appl. Microbiol..

[150] Vignaroli C., Zandri G., Aquilanti L., Pasquaroli S., Biavasco F. (2011). Multidrug-resistant enterococci in animal meat and faeces and Co-transfer of resistance from an Enterococcus durans to a human Enterococcus faecium. Curr. Microbiol..

[151] Agersø Y., Pedersen A.G., Aarestrup F.M. (2006). Identification of Tn5397-like and Tn916-like transposons and diversity of the tetracycline resistance gene tet(M) in enterococci from humans, pigs and poultry. J. Antimicrob. Chemother..

[152] Ramos Y., Morales D.K. (2019). Exopolysaccharide-mediated surface penetration as new virulence trait in *Enterococcus faecalis*. Commun. Integr. Biol..

[153] Ebrahimi A., Schwartzman J., Cordero O.X. (2019). Multicellular behaviour enables cooperation in microbial cell aggregates. Philos. Trans. R. Soc. B Biol. Sci..

[154] Lynch K.M., Zannini E., Coffey A., Arendt E.K. (2018). Lactic Acid Bacteria Exopolysaccharides in Foods and Beverages: Isolation, Properties, Characterization, and Health Benefits. Annu. Rev. Food Sci. Technol..

[155] Salachna P., Mizielińska M., Soból M. (2018). Exopolysaccharide gellan gum and derived oligo-gellan enhance growth and antimicrobial activity in eucomis plants. Polymers.

[156] Abdalla A.K., Ayyash M.M., Olaimat A.N., Osaili T.M., Al-Nabulsi A.A., Shah N.P., Holley R. (2021). Exopolysaccharides as Antimicrobial Agents: Mechanism and Spectrum of Activity. Front. Microbiol..

[157] Sivasankar P., Seedevi P., Poongodi S., Sivakumar M., Murugan T., Sivakumar L., Sivakumar K., Balasubramanian T. (2018). Characterization, antimicrobial and antioxidant property of exopolysaccharide mediated silver nanoparticles synthesized by Streptomyces violaceus MM72. Carbohydr. Polym..

[158] Worsztynowicz P., Schmidt A.O., Białas W., Grajek W. (2019). Identification and partial characterization of proteolytic activity of *Enterococcus faecalis* relevant to their application in dairy industry. Acta Biochim. Pol..

[159] Liu M., Bayjanov J.R., Renckens B., Nauta A., Siezen R.J. (2010). The proteolytic system of lactic acid bacteria revisited: A genomic comparison. BMC Genom..

[160] Nami Y., Bakhshayesh R.V., Jalaly H.M., Lotfi H., Eslami S., Hejazi M.A. (2019). Probiotic properties of enterococcus isolated from artisanal dairy products. Front. Microbiol..

[161] Petrin S., Patuzzi I., Di Cesare A., Tiengo A., Sette G., Biancotto G., Corno G., Drigo M., Losasso C., Cibin V. (2019). Evaluation and quantification of antimicrobial residues and antimicrobial resistance genes in two Italian swine farms. Environ. Pollut..

[162] Ripatti S., Tikkanen E., Orho-Melander M., Havulinna A.S., Silander K., Sharma A., Guiducci C., Perola M., Jula A., Sinisalo J. (2010). A multilocus genetic risk score for coronary heart disease: Case-control and prospective cohort analyses. Lancet.

[163] Zirakzadeh A., Patel R. (2006). Vancomycin-resistant enterococci: Colonization, infection, detection, and treatment. Mayo Clin. Proc..

[164] Santella B., Folliero V., Della Rocca M.T., Zannella C., Pignataro D., Greco G., Montella F., Folgore A., Galdiero M., Galdiero M. (2019). Distribution of antibiotic resistance among *Enterococcus* spp. isolated from 2017 to 2018 at the University Hospital. Int. J. Mol. Clin. Microbiol..

[165] Ayeni F.A., Odumosu B.T., Oluseyi A.E., Ruppitsch W. (2016). Identification and prevalence of tetracycline resistance in enterococci isolated from poultry in Ilishan, Ogun State, Nigeria. J. Pharm. Bioallied Sci..

[166] Chopra I., Roberts M. (2001). Tetracycline Antibiotics: Mode of Action, Applications, Molecular Biology, and Epidemiology of Bacterial Resistance. Microbiol. Mol. Biol. Rev..

[167] Robredo B., Torres C., Singh K.V., Murray B.E. (2000). Molecular analysis of Tn1546 in vanA-containing *Enterococcus* spp. isolated from humans and poultry. Antimicrob. Agents Chemother..

[168] Simjee S., White D.G., McDermott P.F., Wagner D.D., Zervos M.J., Donabedian S.M., English L.L., Hayes J.R., Walker R.D. (2002). Characterization of Tn1546 in vancomycin-resistant Enterococcus faecium isolated from canine urinary tract infections: Evidence of gene exchange between human and animal enterococci. J. Clin. Microbiol..

[169] Ranotkar S., Kumar P., Zutshi S., Prashanth K.S., Bezbaruah B., Anand J., Lahkar M. (2014). Vancomycin-resistant enterococci: Troublemaker of the 21st century. J. Glob. Antimicrob. Resist..

[170] Thumu S.C.R., Halami P.M. (2012). Acquired Resistance to Macrolide-Lincosamide-Streptogramin Antibiotics in Lactic Acid Bacteria of Food Origin. Indian J. Microbiol..

[171] Antibiotics in Animal Farming|Compassion in World Farming. https://www.ciwf.org.uk/research/food-and-human-health/antibiotics-in-animal-farming/.

[172] Terzić-Vidojević A., Veljović K., Begović J., Filipić B., Popović D., Tolinački M., Miljković M., Kojić M., Golić N. (2015). Diversity and antibiotic susceptibility of autochthonous dairy enterococci isolates: Are they safe candidates for autochthonous starter cultures?. Front. Microbiol..

[173] Jahan M., Zhanel G.G., Sparling R., Holley R.A. (2015). Horizontal transfer of antibiotic resistance from Enterococcus faecium of fermented meat origin to clinical isolates of E. faecium and *Enterococcus faecalis*. Int. J. Food Microbiol..

[174] Tran T.H.T., Everaert N., Bindelle J. (2018). Review on the effects of potential prebiotics on controlling intestinal enteropathogens Salmonella and *Escherichia coli* in pig production. J. Anim. Physiol. Anim. Nutr..

[175] Herrero I.A., Fernández-Garayzábal J.F., Moreno M.A., Domínguez L. (2004). Dogs Should Be Included in Surveillance Programs for Vancomycin-Resistant Enterococci. J. Clin. Microbiol..

[176] Manson J.M., Keis S., Smith J.M.B., Cook G.M. (2003). Characterization of a vancomycin-resistant *Enterococcus faecalis* (VREF) isolate from a dog with mastitis: Further evidence of a clonal lineage of VREF in New Zealand. J. Clin. Microbiol..

[177] Peters J., Mac K., Wichmann-Schauer H., Klein G., Ellerbroek L. (2003). Species distribution and antibiotic resistance patterns of enterococci isolated from food of animal origin in Germany. Int. J. Food Microbiol..

[178] Dubin K., Pamer E.G. (2017). Enterococci and Their Interactions with the Intestinal Microbiome. Microbiol. Spectr..

